# Healthy lifestyle index and its association with hypertension among community adults in Sri Lanka: A cross-sectional study

**DOI:** 10.1371/journal.pone.0226773

**Published:** 2020-01-10

**Authors:** Ami Fukunaga, Yosuke Inoue, Nadeeka Chandraratne, Miwa Yamaguchi, Keisuke Kuwahara, Susantha Indrawansa, Nalika Gunawardena, Tetsuya Mizoue, Diyanath Samarasinghe

**Affiliations:** 1 Department of Epidemiology and Prevention, Center for Clinical Sciences, National Center for Global Health and Medicine, Tokyo, Japan; 2 Management, Development & Planning Unit, Ministry of Health, Colombo, Sri Lanka; 3 International Center for Nutrition and Information, National Institute of Health and Nutrition, National Institutes of Biomedical Innovation, Health and Nutrition, Tokyo, Japan; 4 Graduate School of Public Health, Teikyo University, Tokyo, Japan; 5 The Foundation for Health Promotion, Dehiwala, Sri Lanka; 6 Office for Sri Lanka, World Health Organization Country, Colombo, Sri Lanka; 7 Department of Psychological Medicine, University of Colombo, Colombo, Sri Lanka; International University of Health and Welfare, School of Medicine, JAPAN

## Abstract

**Objectives:**

To investigate the association between a healthy lifestyle index (HLI) (i.e., a composite score comprising multiple lifestyle factors) and hypertension among community adults living in Sri Lanka.

**Methods:**

The present study used baseline information of a cluster randomized controlled trial among 456 adults aged 27–65 years in a semi-urban community in Colombo, Sri Lanka. The HLI was constructed by summing a number of low-risk lifestyle factors: low body mass index, sufficient physical activity, non-smoking, low alcohol consumption, and sufficient fruit and vegetable consumption. Hypertension was defined as systolic blood pressure ≥140 mmHg, diastolic blood pressure ≥90 mmHg, or the use of antihypertensive medication. A logistic regression model was used to investigate the association between the HLI (low: 0–2; middle: 3; high: 4–5) and hypertension.

**Results:**

A total of 178 (39%) participants were hypertensive. Compared with the low HLI group, multivariate-adjusted odds ratios (95% confidence intervals) of hypertension were 0.72 (0.44–1.19) and 0.28 (0.15–0.54) for the middle and high HLI groups, respectively (*p-*trend <0.001).

**Conclusions:**

The present study provides essential evidence on an inverse association between adherence to healthy lifestyles and hypertension.

## Introduction

Hypertension is a leading risk factor of cardiovascular diseases (CVDs) [1] and is a major contributor to the global burden of disease [2]. The global prevalence of hypertension in adults increased from 594 million to 1.13 billion from 1975 to 2015 [3]. More importantly, the disease burden associated with hypertension has shifted from high-income countries to low- and middle-income countries (LMICs) [1, 4]. Sub-Saharan Africa, South Asia, and Central and Eastern Europe had the highest blood pressure measurements in 2015 [1]. The global increase in the number of adults with hypertension has been largely contributed by the increase in LMICs.

Several studies in LMICs have identified modifiable risk factors of hypertension, including overweight/obesity, sedentary lifestyle, smoking, alcohol consumption, and low fruit and vegetable consumption [5, 6]. Some LMICs have published hypertension guidelines, which refer to the importance of these lifestyle components in terms of prevention and control of hypertension [7]. Despite these efforts, more research is needed to inform preventive efforts that are cost-effective and easy to implement in LMICs where financial and medical resources are limited.

Accumulating evidence from high-income countries suggests the importance of constructing a healthy lifestyle index (HLI), i.e., a single score based on lifestyle factors that people adopt. Previous studies have revealed a significant association between a HLI and the prevalence [8, 9] or the incidence of hypertension [10–13]. A HLI may capture the combined effects of multiple health-related lifestyles known to co-exist [14, 15]. Given that recent societal changes in LMICs (e.g., urbanization and economic development) may have impacted a wide range of lifestyle factors (e.g., nutritional transition and sedentary lifestyle) simultaneously [5, 16], this topic warrants extensive study in the LMIC context.

Sri Lanka is a LMIC located in South Asia. The gross domestic product increased 10-fold from 8 billion to 87 billion USD from 1990 to 2017 [17]. Along with economic growth, the disease burden due to noncommunicable diseases has been on an increasing trend [3]; high blood pressure was the third largest contributor of disability-adjusted life years in 2017 [18]. Katulanda et al. [19] examined the individual associations among obesity, physical activity, and hypertension in Sri Lanka, but little attempt has been made to investigate the associations between combined multiple healthy lifestyles and hypertension in Sri Lanka as well as in other LMICs.

The purpose of this study was to examine the association between a HLI composed of five modifiable lifestyle factors (body mass index [BMI], physical activity, smoking status, alcohol consumption, and fruit and vegetable consumption) and the prevalence of hypertension among community adults in Sri Lanka.

## Methods

### Study procedure

Data for the present study were derived from a cluster randomized controlled trial that examined the effect of health-promoting activities by trained youth club members on CVD risk among community adults in a semi-urban area of Colombo, Sri Lanka [20]. To recruit study participants, we randomly selected 24 Grama Niladari (GN) divisions, i.e., the rural level administrative division in the country [21]. We then randomly visited 23–29 households in each GN area and enrolled participants between April and August 2016. Trained staff members interviewed the participants about their lifestyle and health using survey questionnaires. Baseline survey was conducted after enrollment and follow-up was conducted one year later in 2017. The present study used information collected at baseline (i.e., before the intervention), which included information on sociodemographic variables, health-related lifestyles, and anthropometric and blood pressure measurements. This study protocol was approved by the Ethics Review Committee of the Sri Lanka Medical Association and the Ethics Committee of the National Center for Global Health and Medicine, Japan. Written informed consent was obtained from all participants prior to the study.

### Participants

A total of 591 community adults were invited for the study, 569 agreed to participate, and 512 completed the baseline survey. We then excluded those with missing data on the second blood pressure measurements (n = 25) and the third blood pressure measurements (n = 18), and those with missing data on selected covariates (n = 13) (described below). As a result, we had an analytical sample of 456 participants (210 men and 246 women) aged 27–65 years (mean, 45.7; standard deviation, 8.2) for the subsequent analysis.

### Construction of the healthy lifestyle index

Information on five modifiable lifestyle factors, BMI, physical activity, smoking status, alcohol consumption, and fruit and vegetable consumption, was used to construct the HLI. Based on previous knowledge and recommendations, we dichotomized each lifestyle factor into a low-risk (adhering to a healthy lifestyle) or a high-risk (not adhering to a healthy lifestyle) group.

Body height and weight were measured to the nearest 0.1 cm and 0.2 kg, respectively, using a portable stadiometer (Seca 213; Seca Inc., Hamburg, Germany) and a digital weighing scale (WB220; Rossmax, Berneck, Switzerland), with participants wearing light clothes and no shoes. BMI was calculated by dividing weight (kg) by height squared (m^2^). We defined the low-risk BMI group as individuals with BMI <25 kg/m^2^ [22]. We defined the low-risk physical activity group as individuals who engaged in ≥150 minutes/week of moderate intensity physical activity or ≥75 minutes/week of vigorous-intense physical activity (including leisure time, occupational, and transportation physical activity) based on the current physical activity recommendations [23]. We defined non-smokers as the low-risk group, including never and former smokers, and the high-risk group as current smokers [24]. The low-risk group for alcohol consumption was defined as ≤2 drinks/day for men and ≤1 drink/day for women based on the Dietary Guidelines in the United States [25], which was also referred in the above-mentioned intervention study in Sri Lanka [20]. Food-based dietary guidelines for Sri Lankans recommend consuming 2–3 servings/day of fruits and 3–5 servings/day of vegetables [26]. Thus, we defined the low-risk group as individuals consuming ≥2 servings/day of fruit and ≥3 servings/day of vegetables.

The low- and high-risk groups received scores of 1 and 0, respectively to compute a composite score ranging from 0 to 5 (Table 1). Because of the small numbers of participants scoring 0 (n = 0), 1 (n = 6), and 5 (n = 8), we categorized the HLI into three groups: 0–2 (low), 3 (middle), and 4–5 (high).

**Table 1:** Components of healthy lifestyle index.

Healthy lifestyle index components	Low-risk group (score 1)	High-risk group (score 0)
Body mass index	<25 kg/m^2^	≥25 kg/m^2^
Physical activity	≥150 minutes/week of moderate-intensity physical activity or ≥75 minutes of vigorous-intensity physical activity	<150 minutes/week of moderate-intensity physical activity and <75 minutes of vigorous-intensity physical activity
Smoking status	Non-smoker (never or former)	Current smoker
Alcohol consumption	≤2 drinks/day for men ≤1 drink/day for women	>2 drinks/day for men >1 drink/day for women
Fruit and vegetable consumption	Fruit consumption ≥2 servings/day and vegetable consumption ≥3 servings/day	Fruit consumption <2 servings/day and/or vegetable consumption <3 servings/day

### Assessment of hypertension

Trained staff members performed blood pressure measurements using an automatic blood pressure monitor (UA-621; A&D, Tokyo, Japan). Blood pressure was measured three times with participants seated and their right arm supported at the heart level. Participants were instructed to rest for 10 minutes in a quiet room prior to measurement. The last two measurements were used to calculate mean systolic and diastolic blood pressure, and hypertension was defined as systolic blood pressure ≥140 mmHg, diastolic blood pressure ≥90 mmHg, or the use of antihypertensive medication.

### Covariates

We collected information on covariates, including age (years, continuous), sex (men or women), education (primary school, junior high school, or high school or higher), household income (<25,000, 25,000–40,000, or >40,000 Rupees/month), current worker (yes or no), adding salt when cooking rice (always, often, sometimes, rarely, or never), and history of diabetes (yes or no) via a questionnaire.

### Statistical analysis

We determined frequencies and means of the participants’ characteristics stratified by hypertensive status. Differences in the characteristics between participants with and without hypertension were assessed using the *t*-test (continuous variables) or the chi-squared test (categorical variables). To examine the association between the HLI and hypertension, we performed multiple logistic regression analysis and calculated odds ratios (ORs) and corresponding 95% confidence intervals (CIs) of hypertension. We adjusted for age and sex in model 1. In model 2, we additionally adjusted for education, household income, current working status, adding salt when cooking rice, and history of diabetes. Given the cluster sampling procedure used in this study, we accounted for GN as a cluster in the models.

In addition, to examine the individual associations of the five lifestyle factors with hypertension, we performed multiple logistic regression analysis in which we incorporated the lifestyle factors as independent variables in the models. We used the same steps for adjustment as in the main analysis.

A *p*-value <0.05 was considered significant (two-tailed). All statistical analyses were performed using SAS version 9.4 software (SAS Institute, Cary, NC, USA).

## Results

Table 2 shows the participants’ characteristics stratified by hypertensive status. We identified 178 participants with hypertension (39%). Those with hypertension were more likely to be older, male, and have a history of diabetes.

**Table 2:** Characteristics of the study participants from a semi-urban community in Colombo, Sri Lanka, 2016 (n = 456).

	All (n = 456)	No hypertension (n = 278)	Hypertension (n = 178)	*p*-value
**Covariates**				
Age, mean [SD]	45.7 [8.2]	44.1 [8.3]	48.1 [7.4]	<0.0001
Sex (men), *n* (%)	210 (46.1)	116 (41.7)	94 (52.8)	0.02
Education, *n* (%)				0.93
Primary school (grade 1–5)	65 (14.3)	41 (14.8)	24 (13.5)	
Junior high school	274 (60.1)	166 (59.7)	108 (60.7)	
High school or higher	117 (25.7)	71 (25.5)	46 (25.8)	
Household income (Rupees/month), *n* (%)				0.27
<25,000	215 (47.2)	128 (46.0)	87 (48.9)	
25,000–40,000	165 (36.2)	108 (38.9)	57 (32.0)	
>40,000	76 (16.7)	42 (15.1)	34 (19.1)	
Current worker (yes), *n* (%)	244 (53.5)	155 (55.8)	89 (50.0)	0.23
Adding salt when cooking rice (always or often), *n* (%)	223 (48.9)	132 (47.5)	91 (51.1)	0.45
History of diabetes (yes), *n* (%)	75 (16.5)	29 (10.4)	46 (25.8)	<0.0001
**Blood pressure measurements**				
Systolic blood pressure (mmHg), mean [SD]	125.7 [19.9]	115.2 [11.0]	142.1 [19.7]	<0.0001
Diastolic blood pressure (mmHg), mean [SD]	83.2 [12.1]	76.7 [7.2]	93.4 [11.1]	<0.0001
**Healthy lifestyle index components**				
Body mass index (<25 kg/m^2^), *n* (%)	237(52.0)	172 (61.9)	65 (36.5)	<0.0001
Physical activity (≥150 minutes/week of moderate-intensity or ≥75 minutes/week of vigorous-intensity physical activity), *n* (%)	381 (83.6)	238 (85.6)	143 (80.3)	0.14
Smoking status (never or former), *n* (%)	385 (84.4)	236 (84.9)	149 (83.7)	0.73
Alcohol consumption (no or moderate amount^+^), *n* (%)	427 (93.6)	264 (95.0)	163 (91.6)	0.15
Fruit consumption (≥2 servings/day) and vegetable consumption (≥3 servings/day), *n* (%)	18 (4.0)	9 (3.2)	9 (5.1)	0.33

Abbreviation: SD, standard deviation.

^+^Moderate amount refers to ≤2 drinks/day for men and ≤1 drink/day for women.

Table 3 shows the results of the logistic regression analysis investigating the association between the HLI and hypertension. A significant association was observed between the HLI and hypertension; the ORs (95% CI) of hypertension were 0.72 (0.43–1.20) and 0.29 (0.17–0.51) for the middle and high HLI groups, respectively (*p-*trend < 0.0001) in model 1. When the model was further adjusted for sociodemographic variables, current working status, adding salt when cooking rice, and history of diabetes, the corresponding figures were 0.72 (0.44–1.19) and 0.28 (0.15–0.54) for the middle and high HLI groups, respectively (*p-*trend <0.001) (model 2).

**Table 3:** Odds ratios and 95% confidence intervals of hypertension according to the healthy lifestyle index categories.

	Healthy lifestyle index			
	0–2 (low) (n = 97)	3 (middle) (n = 184)	4–5 (high) (n = 175)	*p*-trend*
Cases, n (%)	50 (51.6)	82 (44.6)	46 (26.3)	
Model 1^a^	1.00 (reference)	0.72 (0.43–1.20)	0.29 (0.17–0.51)	<0.0001
Model 2^b^	1.00 (reference)	0.72 (0.44–1.19)	0.28 (0.15–0.54)	<0.001

*Based on multiple logistic regression analysis incorporating healthy lifestyle index as a continuous variable.

^a^Model 1 is adjusted for age (years, continuous) and sex (men or women).

^b^Model 2 is adjusted for variables in model 1, and education (primary school, junior high school, or high school or higher), household income (<25,000, 25,000–40,000, or >40,000 Rupees/month), current worker (yes or no), adding salt when cooking rice (always, often, sometimes, rarely, or never), and history of diabetes (yes or no).

Associations between individual healthy lifestyle factors and hypertension are shown in Table 4. Only BMI was significantly associated with hypertension (OR = 0.27; 95% CI = 0.15–0.49).

**Table 4:** Individual associations between healthy lifestyle index components and hypertension.

	Model 1^a^	Model 2^b^
Healthy lifestyle index components	OR (95% CI)	OR (95% CI)
Body mass index (<25 kg/m^2^)	0.28 (0.17–0.46)*	0.27 (0.15–0.49)*
Physical activity (≥150 minutes/week of moderate-intensity or ≥75 minutes/week of vigorous-intensity physical activity)	0.76 (0.40–1.45)	0.72 (0.40–1.31)
Smoking status (never or former)	1.26 (0.83–1.93)	1.39 (0.89–2.17)
Alcohol consumption (no or moderate amount^+^)	0.55 (0.23–1.27)	0.47 (0.18–1.23)
Fruit consumption (≥2 servings/day) and vegetable consumption (≥3 servings/day)	1.43 (0.39–5.21)	1.41 (0.39–5.20)

*p-value<0.001.

^+^ Moderate amount refers to ≤2 drinks/day for men and ≤1 drink/day for women.

^a^Model 1 is adjusted for age (years, continuous) and sex (men or women).

^b^Model 2 is adjusted for variables in model 1, and education (primary school, junior high school, or high school or higher), household income (<25,000, 25,000–40,000, or >40,000 Rupees/month), current worker (yes or no), adding salt when cooking rice (always, often, sometimes, rarely, or never), and history of diabetes (yes or no).

## Discussion

In this cross-sectional study, the HLI, which was calculated based on five lifestyle parameters (BMI, physical activity, smoking status, alcohol consumption, and fruit and vegetable consumption), was significantly associated with a lower prevalence of hypertension among community adults in Sri Lanka. More specifically, the OR for hypertension was 0.28 among those in the high HLI group (score = 4–5), compared to those in the low HLI group (score = 0–2).

The inverse association between the HLI and hypertension observed in our study has been previously reported in high-income countries including Australia [8, 10], USA [11–13], and Ireland [9]. One such example is an Irish cross-sectional study [9] that used a similar HLI and showed that participants with four or more healthy lifestyle factors (i.e., low BMI, absence of central obesity, physically active, non-smoking, low alcohol consumption, and healthy dietary pattern) had a significantly lower prevalence of hypertension compared with those with 0–1 healthy lifestyle factor. Our study extends these previous studies by showing the association between the HLI and hypertension in the LMIC context, where disease burden associated with hypertension is increasing.

Lifestyle factors used to construct HLIs vary across studies. BMI, physical activity, smoking status, and alcohol consumption are frequently employed [8–13], whereas other factors are used only occasionally, including sedentary time [8], sleep duration [8], waist-hip ratio [9], psychological distress [10], cardiorespiratory fitness [12], and the use of medication (non-narcotic analgesics [11, 13] and supplemental folic acid [13]). Several studies have incorporated dietary components to construct the HLI, but with different measures, e.g., fruit and vegetable consumption [10] and adherence to the Dietary Approaches to Stop Hypertension (DASH) diet [11, 13], which was designed to lower blood pressure. Despite the heterogeneity in the components of the HLIs, the present study and previous studies provide robust evidence for the association between the HLIs and hypertension.

When we investigated the individual associations of the five lifestyle factors with hypertension, only BMI revealed a significant association. This finding is similar to those of previous HLI studies that examined the associations between individual factors and hypertension [9–13]. This result also accords with those reported by Andriole et al. [27], who investigated how combinations of six modifiable lifestyle factors (i.e., BMI, waist circumference, physical activity, smoking, alcohol consumption, and diet) are differentially linked with the incidence of hypertension in a large German cohort, and found that being overweight or obese (BMI ≥25 kg/m^2^) contributed the most to hypertension risk among the six lifestyle factors. The present study adds to evidence that obesity plays a major role in the development of hypertension.

We did not find evidence for significant associations between the individual HLI components, other than BMI, and hypertension, but this does not mean that we do not need to account for these factors, as the lowest prevalence of hypertension was observed when we accounted for all of the components. Previous studies have also considered the combined effect of multiple health-related lifestyles, not all of which were significantly associated with the outcome [9, 10, 12]. The present study, along with previous studies, suggests that the HLI is a composite score that shows the extent to which an individual leads a healthy lifestyle as a whole.

Several limitations of the present study should be mentioned. First, the cross-sectional design of the study prevented us from determining the direction of the association and inferring a causal association. Second, the small sample size may have limited the ability to detect the associations between individual lifestyle factors and hypertension. Third, although we adjusted for numerous potential confounders, we could not rule out the possibility that the observed associations were due to residual confounders or unmeasured confounders (e.g., family history of hypertension). Fourth, we did not have information on the amount of sodium consumed, which has a significant effect on blood pressure [28]. Finally, our findings may not be generalizable to urban or rural communities in Sri Lanka or the country as a whole, as our participants were drawn from a semi-urban community. Therefore, caution is required when generalizing the findings.

## Conclusions

The present study suggests a significant association between greater adherence to healthy lifestyles (i.e., BMI <25 kg/m^2^, sufficient physical activity, non-smoking, low alcohol consumption, and sufficient fruit and vegetable consumption) and a lower prevalence of hypertension among community adults in Sri Lanka.
